# Low geomagnetic field strength during End-Cretaceous Deccan volcanism and whole mantle convection

**DOI:** 10.1038/s41598-020-67245-6

**Published:** 2020-07-01

**Authors:** Radhakrishna T., Asanulla R. Mohamed, Venkateshwarlu M., Soumya G. S.

**Affiliations:** 1 National Centre for Earth Science Studies, Trivandrum, 695011 India; 2 CSIR-National Geophysical Research Institute, Hyderabad, 500 007 India; 3 Present Address: GITAM University, Nagadenehalli, Doddaballapur Taluk, Bengaluru, 561203 India; 4 Present Address: Department of PG Studies and Research in Geology, Government College Kasaragod, Vidyanagar, 671123 India

**Keywords:** Solid Earth sciences, Geodynamics, Palaeomagnetism

## Abstract

Knowledge about long-term variation of the geomagnetic dipole field remains in its nascent stage because of the paucity of reliable experimental data over geological periods. Here, we present the first robust experimental data from the largest Cretaceous flood basalt province on Earth, the ~65–66 Ma Deccan basalt within a thick (1250 m) unbiased stratigraphic section down to the basement, recovered from a drill hole of the Koyna Deep Scientific Drilling Project in the Western Ghats, India. Critical analysis of the result along with similar results of the Cretaceous age find that (i) the dipole moment during the end Cretaceous Deccan eruption is the lowest in whole of Cretaceous (ii) dipole moment at the onset/termination of the Cretaceous Normal Superchron is apparently lower relative to that in mid-superchron, however, such differences cannot be deciphered in shorter polarities probably because of insufficient time to develop recognizable variations (iii) inverse relation between dipole moment and reversal rate is lacking and (iv) a cause and effect relation between core-mantle boundary heat flux and low dipole moment that appears to be the principle governing factor in forming the Large Igneous Provinces on the surface of earth.

## Introduction

Long-term variability of Earth’s early Dipole magnetic field (palaeointensity; PI) is a complex internally driven phenomenon. Numerical simulations apart, geological materials are the only source for direct experimental estimates. However, large failure rate, prolonged duration of experiments and increased complexities in deciphering magnetic field at early periods have become a serious hindrance for this experimental approach; therefore, a vast number of studies have focused over the period of one million year, where the determinations are relatively more straight forward. Yet, in recent years, there have been compilations of PI data over the geological period with an objective to develop a robust global database (for example, PINT15 database: http:// earth.liv.ac.uk /pint/).

**Figure 1:**
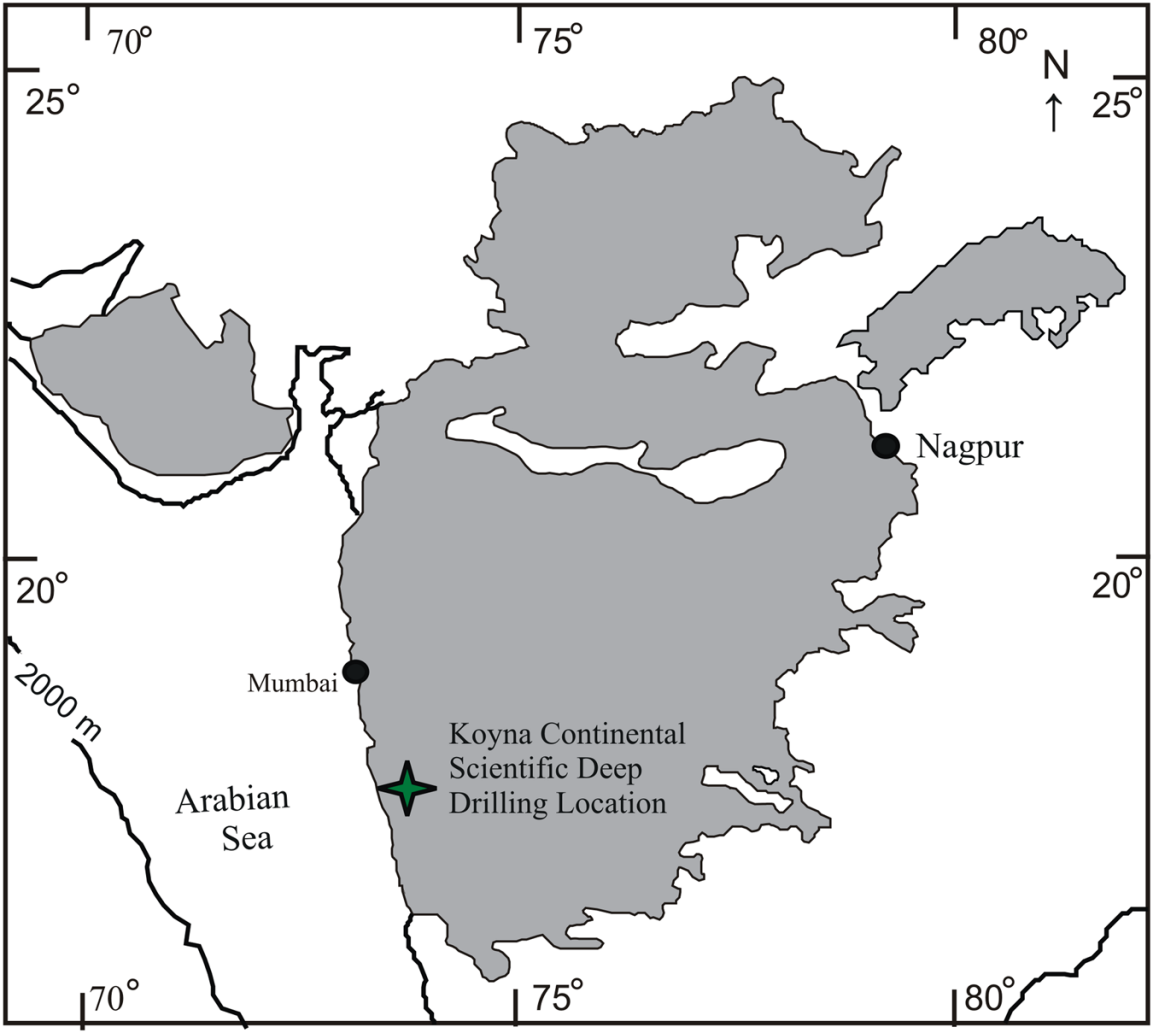
Geological map showing areal spread of the Deccan flood basalt eruptions and location of the Koyna Continental Scientific Deep  Drilling Project drill hole (KBH-7) of this study.

The Mesozoic period has drawn a special attention with the proposal of a period of relatively low field, one-third of the Cenozoic value, known as the Mesozoic Dipole Low (MDL)^1^. Whereas some investigations argue that the MDL proposal is not tenable^2–6^, the hypothesis has gained wider support^7–9^ although many disagreements exist on the duration of the low field. The MDL was suggested to confine to the Jurassic Quiet Zone (~145–165 Ma)^10^, and some others extend it into early Cretaceous^11–13^.The low field strength is also variably described with respect to the Cretaceous Normal Superchron (CNS); some argue that the MDL extended into the CNS^14,15^, some others, mostly from China, report a low field at the onset of CNS^16–18^ and some works find it at the end of the CNS^19–22^. Likewise, the low fields are correlated with high reversal frequency^9,23–26^, but there are arguments decoupling the two^21,27,28^. The fluctuation in geomagnetic field strength is intricately linked to core-mantle boundary (CMB) heat flow and whole mantle convection processes^25,29–32^. In light of the ongoing hot debate and the large-scale geodynamic significance, we conducted a comprehensive PI study on one of the prominent surface manifestations of whole mantle convection, the Deccan flood basalt covering an unbiased stratigraphic section within a thick drill hole of the Continental Scientific Deep Drilling Project in the Koyna region of the Western Ghats, India (Fig. 1). We combine the results with other high quality Cretaceous global data and interpret in terms of relationships between geomagnetic behavior, polarity reversals and deep mantle processes.

## Materials and Methods

The Koyna Deccan basalt drill hole (KBH-7) samples of the present PI study come from nineteen flows comprising of 1250 m thick lava sequence down to the Archaean gneissic basement. The lava pile represents the stratigraphic formations that constitute the Wai Group. The top ~350 m section belongs to the Mahabaleswar formation with normal polarity and thereafter, the section down to the basement is monotonously of reverse polarity that represents the Ambenali, upper Poladpur and lower Poladpur formations in succession downwards^33^. The drill hole section recovered at least seven red boles amounting to a thickness of more than 5 m and a few alteration zones indicating that smaller pulses of eruptions are separated by longer times of volcanic quiescence. The time needed to form the red bole estimated a few kilo years of quiescence^34^. This suggests that several kilo years to millennia are recorded in our PI data. Recent high precision U-Pb and ^40^Ar/^39^Ar ages are an added advantage for the present study; these results unequivocally place the age of the Koyna Deccan drill hole succession at ~65–66 Ma^35,36^.

PI experimental techniques and the reliability criteria have been revolutionized with pronounced modifications over the years, although the Thellier–Thellier^37^ method remains more fundamental for most of the investigations. Many of the earlier results have some limitations and highlight the need for additional accurate data^28^. The PI study of the well-dated Deccan section adopted a systematic approach: determined detailed rockmagnetic properties as a prelude to PI experiments, used a more accepted thermal PI method (Zero field-In field)^38^, conducted partial thermoremanent magnetization (pTRM) checks and pTRM tail checks, assessed for cooling rate and anisotropy corrections and followed standard set of strict reliability and quality criteria to consider results to compute field strength at the time (c. 65–66 Ma) of the Deccan flood basalt eruption (see details of methods in electronic supplement).

## Results

Although the investigations were performed on 76 samples covering the 19 flows down to the basement, only 34 samples from nine flows were found successful in yielding PI data. A judicious selection, considering that (i) at least there are successful determinations from three independent samples (ii) the samples show within flow consistency that is within limits of standard deviation (iii) the uncertainties over the mean value of the flow are less than 20% and (iv) none of the flows represent transition field, eight lava flows (26 samples) provide a reliable mean palaeointensity of 7.30 ± 3.45μT. Using the remarkably well constrained Deccan palaeopole (37.8°N, 282.6°E)^34^, ~65 Ma palaeolatitude for the Koyna drill hole is estimated as 28.1°S to calculate the Virtual Dipole Moment (VDM),which is independent of the latitude. The calculated mean VDM is 1.46 ± 0.69 × 10^22^ Am^2^ (range: 2.19–0.28 ×10^22^ Am^2^; see Supplementary Table S1). Comparable values were estimated on surface samples of the Deccan (unpublished) in an independent study funded by the Indo-French Collaboration for Promotion of Advanced Research (IFCPAR). Because (a) the magnetization directions from cooling units of the formations sampled in this study show that secular variation has been averaged^39^ and (b) geologically an interval of kilo years to million years long has been sampled, the mean paleointensity is accepted to represent a paleomagnetic dipole moment, rather than a snapshot dipole moment. Thus, this result is the best dipole moment result from one of the two largest Phanerozoic flood basalt provinces of the world and the largest Cretaceous igneous province.

## Discussion

Mesozoic dipole moments have been reported from volcanic rocks and deep sea sediments. Now, it is well known that sediment record is smoothed over time and provides relative intensity record and thus provides a continuous evolution of palaeofield strength rather than absolute PI values^40^. Furthermore, current methods to estimate absolute PI are applicable only to materials that acquired a thermal remanent magnetization by cooling in the Earth’s magnetic field and volcanic rocks are the best choice to provide absolute estimates on millions and perhaps billions of years scale^41^. Not all volcanic rocks of PI investigation are constrained for their ages by radiometric methods. Also, some of the PI determinations come from submarine basaltic glass in which the magnetic minerals are sometimes chemically altered during PI experiments, and thus highlight the need to obtain the data from magnetically stable volcanic rocks to clarify the nature of geomagnetic field in the Late Cretaceous^21,28^. Furthermore, most submarine basalt glass values represent averages of data from individual samples and not the averages of independent time units constrained by lava flow stratigraphy and paleomagnetic directional analysis^23,28^. In these respects, the Koyna drill hole samples of the Deccan are a valuable resource material because these are fresh subaerial eruptions with high precision age constraints and comprise several cooling units in an unbiased stratigraphic section down to the basement. Based on all these factors, we consider here the Deccan results in combination with other Cretaceous PI results from radiometrically dated igneous rocks and obtained by the Thellier method with pTRM checks, excluding submarine basalt glass data.

**Figure 2:**
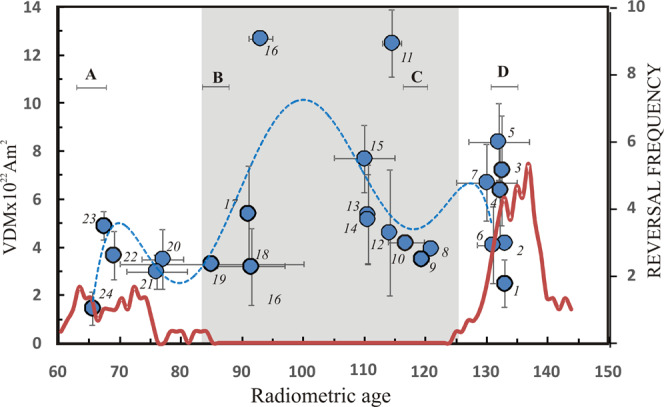
Distribution of VDM data during the Cretaceous period. The data set is as listed in table S-T2. Note a polynomial trend showing high dipole moment with low values on either end of the CNS. The numbers next to the data points are linked to the serial numbers in table S-T2. Also shown are the timing of mantle plumes of the Cretaceous responsible for the major large igneous provinces marked as A, B, C and D: A denotes the Reunion related Deccan flood basalt (and near synchronous ~62 Ma North Atlantic Tertiary Volcanic Province), B denotes the Marion plume related Madagascan igneous province (and near synchronous ~ 90 Ma Ontong Java plateau), C denotes the Kerguelen plume related Rajmahal volcanism and D denotes the Tristan da Cunha plume related Parana basalts. The ages of these LIPs are shown with error bars. It can be seen the low dipole moments correspond to the mantle plumes/LIPs in age. Area coverage of LIPs in each age bracket (A: >3; B: <3.5; C: ~2 and D: ~1.5 in million km^2^)^49,50^ may indicate a measure of their magnitudes. Polarity reversal frequency with 3 Ma running average from the 2012 GPTS^51^ is shown in red line. The grey shade demarcates the CNS.

The data meeting the above criteria are listed in Supplementary Table S2 and are illustrated in Fig. 2. Most striking is that the mean VDM value of the Deccan is the lowest among whole of the Cretaceous data. The combined VDM results of the Cretaceous are fitted by a polynomial trend in Fig. 2. We forced the polynomial fit to pass through our data point 24 because of its robust estimation. The trend reflects high field strength in the mid-CNS and relatively low fields on either end of the CNS. Although data are sparse for the mid-CNS period, the stronger dipole in the mid-CNS is supported by numerical models predictions^31,32^. The low VDM values during the onset and termination of the superchron are interpreted here to suggest that the polarity transitions are marked by relatively lower dipole field strength. That is, the geodynamo becomes more turbulent resulting in low VDM values while approaching a reversing regime and attains gradually a non-reversing state with high VDMs in the mid-CNS interval. Differences in the VDM fluctuations across polarity intervals of shorter time span could be so low that cannot be resolved by the present PI experimental protocols, however, should be detected on superchron scales. Studies with respect to the Permian Kiaman Long Reversed Superchron (~265–310 Ma) and the Moyero Reversed Superchron (~460–490 Ma) will be more promising to test the idea proposed here; however, reliable PI results are not available for these time frames. This study underlines the urgent need of PI investigations for these two superchron time frames.

The dipole moment variations have been linked to reversals frequency based on observed low dipole moments during the high reversal frequency middle Jurassic period (~145–65 Ma) and high dipole moments during the CNS^9,23,25,26,29,42^. The high field strength during mid-CNS and low values while entering into and coming out of CNS may be viewed as an indication for inverse relation between field strength and reversal frequency. If there is such an inverse relationship between reversal rate and dipole moment, then the dipole moment at the time of the Deccan eruption should be higher than that during the early Cretaceous (~130–135 Ma) when the reversal rate is more than double to that of the Deccan times (Fig. 2) and also it should be higher than that during mid-Jurassic and in the late Cenozoic when the reversal rates are high (>10 and 5 per Ma respectively). In sharp contrast, the VDM value determined for the Deccan eruption is lower than that of ~130–135 Ma (Fig. 2) and much lower than that in mid-Jurassic (for ex., a VDM of 3.27 ± 0.49 ×10^22^Am^2^ at 166 Ma^9^) and the late Cenozoic (for ex., ~5×10^22^Am^2^ in Miocene^43^); thus the comparison does not support inverse relation between reversal frequency and dipole moment at all times despite the coincidence of low dipole moments and high reversal frequency during mid-Jurassic^10,25,44^ and Devonian^45,46^.

Numerical dynamo models^25,26,29,31,32,42,47^ predict a relationship between high CMB heat flux, via large-scale mantle convection, low dipole moments and high reversal frequency. We find that coupling between dipole moments and CMB heat flux appears to be evident. The low dipole moment is coincident with increased heat flow generated by hyperactive core-mantle process. In consequence, mantle plume departs from the CMB resulting in significant crustal growth at the surface by addition of huge volumes of mantle inputs that manifest in the form of large igneous provinces. The best examples are the Reunion and Marion plume inputs corresponding to the low dipole moments at the end of (and also following) the CNS; similarly the Tristan da Cunha and Kerguelen plume inputs correspond to the low fields at the onset of CNS. However, the high VDM value of the Kerguelen plume related Rajmahal traps (Supplementary Table S2 and Fig. 2) might indicate some time lag between the plume initiation at the CMB and its surface eruption^32^. The mid-Jurassic low dipole moment can be ascribed to high CMB heat flow and crustal growth linked to the Conrad mantle plume activity represented by the Karro igneous province comprising high reversal frequency (at least seven polarity transitions within one Ma)^48^. Deccan flood basalt is the largest igneous outpouring in the whole of Cretaceous and correspondingly the CMB was more hyperactive and resulted in the lowest dipole moment in the entire Cretaceous. Conversely, the mid superchron period (~95–115 Ma) is a period of near quiescence for CMB heat flow and thus mantle plume activity was lowest and high dipole moment developed. Thus our results support the coupling between the dipole moment and CMB heat flow proposed by the numerical models, but suggest that the links with reversal frequency still remain elusive and probably another ingredient also controlling reversal frequency.

## Conclusion

This paper presents the first robust palaeointensity experimental data from the successive Deccan basalt lava flows of end Cretaceous age (~66–65 Ma) within a continuous drill core section of the Continental Scientific Deep Drilling Project in the Western Ghats, India. Eight lava flows provide a reliable mean VDM value (1.46±0.69 ×10^22^ Am^2^). The dipole moment is clearly time-averaged because the mean value is estimated using data from undisputed multiple cooling units of the fresh subaerial lava flows in an unbiased thick stratigraphic section down to the basement, covering kilo years to more than a million year time frame. The results highlight (a) Dipole moment during the end Cretaceous Deccan eruption is the lowest in the whole of Cretaceous while dipole moment is generally lower at onset/termination of Cretaceous Normal Superchron relative to mid-superchron times (b) lack of perfect inverse relation between dipole moment and field reversal rate in contrast to many studies invoking coupling between the two and (c) a cause and effect relationship between CMB heat flux and the dipole low, supporting the predictions of the numerical models; large igneous provinces are shown as manifestations of this activity on the surface of earth.

## Supplementary information

Supplementary information.

## Data Availability

All data generated or analyzed during this study are included in this published article (and its Supplementary Information files).
