# Supplementary material for: Low geomagnetic field strength during End-Cretaceous Deccan volcanism and whole mantle convection
Source: Sci Rep. 2020 Jul 1;10:10743. doi: 10.1038/s41598-020-67245-6 (PMC7329830; doi:10.1038/s41598-020-67245-6)
Supplement: Supplementary file 1 — Supplementary information. [file 41598_2020_67245_MOESM1_ESM.docx]

**Supplementary information**

Low geomagnetic field strength during end Cretaceous Deccan volcanism and whole mantle convection

Radhakrishna, T^1*^., Asanulla R. Mohamed ^1^., Venkateshwarlu M^2^ and Soumya G.S. ^1^

^1^National Centre for Earth Science Studies, Trivandrum 695011,India

^2^CSIR National Geophysical Research Institute, Hyderabad 500 007,India

*corresponding author: Email:tradha1@rediffmail.com

Rockmagnetism: Rockmagnetic experiments were conducted on at least one samples from each eruptive lava flow recovered within the Koyna Drill hole Deccan basalt section as a prelude to Palaeointensity experiments. The rockmagnetic characterisation was done in terms of Natural Remanent Magnetization, magnetic susceptibility, An-hysteretic remanence magnetization, Isothermal remanent magnetization, Lowrie-Fuller test, hysteresis properties and thermomagnetic (Curie experimental) heating-cooling cycle results. The results are consistent with a titanomagnetite carrier with SD and PSD character and about half the number of flow samples show perfect or a little widely spaced reversible thermomagnetic curves that may be promising for PI determinations. The samples possess minor viscous component and alternating field demagnetizations have randomized this viscous component. Hence samples of PI investigation were subjected to prior alternating field demagnetizations to remove viscous component. Standard thermal demagnetization of samples shows similar behavior to that described in previous studies (1-3). Complete set of results are described in a separate paper.

PI experiments and reliability criteria: Thermal PI measurements were performed using the Coe (5) modification of the original Thellier and Thellier (4) double heating protocol. Zero-field and in-field (ZI) heatings were carried out using MMTD-1Thermal Demagnetizer (Magnetic Measurements, UK) and their remanence values were measured with a JR-6 dual speed spinner magnetometer (AGICO, CZECH Republic). The ZI temperature steps were employed in 50°C steps up to 350°C and thereafter 25°C steps up to 600°C. For the infield steps, a small laboratory field of 45µT was applied along the Z-direction of the samples and the samples were always kept in the same position and orientation throughout the ZI experiments. The partial thermoremanent magnetisation (pTRM) and pTRM tail checks were performed at least in four selected steps between 150-500°Cto evaluate the reliability of a PI estimate and multi-domain nature of samples. pTRM tail test was carried out following the procedures described by Riisager and Riisager (6). Cooling rate dependence of TRM was investigated on all samples following a modified procedure described by Chauvin et.al^7^. Anisotropy of anhysteretic remanent magnetization (AARM) was also measured in six directions (+X,-X,+Y,-Y,+Z and -Z) using Selkin et al (8) procedure to monitor Possible bias due to anisotropy effect. The results indicate that the PI values are little influenced by either of the two. Hence, the raw PI data are plotted in Arai-Nagata plots and analysed with Thellier tool software (9). PI values at sample level are accepted when (i) the NRM-TRM direction displays uni-vectoral behaviour projecting to origin on orthogonal plots with maximum angular deviation is ≤6° (ii) linear segment on the Arai-Nagata plot is defined by >7 points (iii) fraction of NRM value ≥ 0.4 (iv) straightness of the selected data points (*β*) is less than 0.12 and (v) the pTRM checks are positive within 10% and (vi) the Quality factor (q) is >5. Representative sample data are shown as typical example in Supplementary Fig S1 and individual sample results are given in S Supplementary Table S1.

Altogether 34 samples from nine lava flows of the Deccanbasalt within the drill hole have given the PI values (see Supplementary Table S1), but Six lava flows (#8, 14, 16, 19, 22 and 24; 19 samples) passed the above reliability criteria. Their PI values range between 1.39 and 10.94 μT with a mean value of 6.90±3.79 μT. However, the PI values of one sample from flow #1 (1b1) and two samples from flow 9 (9b and 9b2) have obtained marginally lower q value, but satisfy all reliability criteria; the PI values of these three samples and the mean PI values of these two flows, by including these samples, are well within uncertainties of the above mean. Accordingly, the flows #1 and 9 are also taken into account for estimating the mean PI value of the Deccan. The mean PI value so calculated (7.30±3.45μT; N=8 flows) is indistinguishable from the mean of 6 flows and there is no reason to discard values of these two flows. Thus 7.30±3.46μT is considered as the mean PI estimate for the ~65-66 Ma Deccan basalt. All the eight flow PI data meet the judicious reliability criteria set in this paper (see the main text) to calculate palaeomagnetic dipole moment. Hence mean virtual dipole moment value of the Deccan is calculated from the PI data of the eight flows and compiled along with other near comparable volcanic rocks which radiometric ages are available (Supplementary Table S2).

**References for supplementary**

1. D. Vandamme, V. Courtillot, J. Besse, R. Montigny, Paleomagnetism and age determinations of the Deccan Traps (India): Results of a Nagpur-Bombay traverse and review of earlier work: Rev. Geophys. **29**, 159–190, doi: 10.1029/91RG00218 (1991).
2. A.L. Chenet, et al., Determination of rapid Deccan eruptions across the Cretaceous–Tertiary boundary using paleomagnetic secular variation: 2. Constraints from analysis of eight new sections and synthesis for a 3500-m-thick composite section: J Geophys. Res. **114**, 38 doi: 10.1029/2008JB005644 (2009).
3. A.E Jay, C. Mac Niocaill, M.Widdowson, S. Self, W. Turner, New Paleomagnetic data from the Mahabaleshwar Plateau, Deccan flood basalt province, India: Implications for the volcanostratigraphic architecture of Continental flood basalt province. Jour. Geol. Soc. London, **122,** 13-24 (2009).
4. E. Thellier, O. Thellier, SUR I’intensite du champ magnetiqueterrestredans le passé historiqueetgeologique. Ann Géophys. **15**, 285–376 (1959).
5. R.S. Coe, Paleointensities of the Earth’s magnetic field determined from Tertiary and Quaternary rocks. J Geophys. Res. **72(12):**3247–62 (1967).
6. P. Riisager, J. Riisager, Detecting multidomain magnetic grains in Thellier palaeointensity experiments, Phys. Earth Planet. Inter. **125**, 111–117 (2001).
7. A. Chauvin, Y. Garcia, Ph. Lanos, F. Laubenhaimer, Paleointensity of the geomagnetic field recovered on archaeomagnetic sites from France, Phys. Earth Planet. Inter*.* **120**, 111–136 (2000).
8. P. A. Selkin, J.S. Gee, L .Tauxe, W.P. Meurer, A.J. Newell, The effect of remanence anisotropy on paleointensity estimates: a case study from the Archean Stillwater Complex, Earth Planet. Sci. Lett*.* **183**(3–4), 403–416 (2000).
9. R. [Leonhardt](https://agupubs.onlinelibrary.wiley.com/action/doSearch?ContribAuthorStored=Leonhardt%2C+R), C. [Heunemann](https://agupubs.onlinelibrary.wiley.com/action/doSearch?ContribAuthorStored=Heunemann%2C+C),   D. [Krása](https://agupubs.onlinelibrary.wiley.com/action/doSearch?ContribAuthorStored=Kr%C3%A1sa%2C+D), Analyzing absolute paleointensity determinations: Acceptance criteria and the software Thellier Tool 4.0. [Geochemistry, Geophysics, Geosystems](https://agupubs.onlinelibrary.wiley.com/journal/15252027) [**5,**](https://agupubs.onlinelibrary.wiley.com/toc/15252027/2004/5/12) (2004).
10. S. C. Dodd, A.R. Muxworthy, C.M. Niocaill, Paleointensity determinations from the Etendekaprovince, Namibia, support a lowmagnetic field strength leading up to the Cretaceous normal superchron. Geochem. Geophys. Geosyst. **16**, 785– 797, doi:10.1002/2014GC005707 (2015).
11. V. V. Shcherbakova, B. Z. Asanidze, V. P. Shcherbakov, G. V. Zhidkov. Geomagnetic field paleointensity in the Cretaceous from Upper Cretaceous rocks of Georgia. Izv. Phys. Solid Earth. **43(11)**, 951–959 (2007).
12. W. Kim, S.J Doh, Y. Yu. Reliable paleointensity determinations from Late Cretaceous volcanic rocks in Korea with constraint of thermochemical alteration. Phys. Earth Planet. Inter.**279**, 47–56 (2018).
13. B., Chang, W., Kim, S.J Doh, Y Yu, Paleointensity determination of Late Cretaceous basalts in northwest South Korea: implications for low and stable paleofield strength in the Late Cretaceous. Earth Planets Space. **65**, 1501–1513 (2013).
14. R.S. Coe, S. Gromm ´e E.A. Mankinen,. Geomagnetic paleointensities from radiocar- bon-dated lava flows on Hawaii and the question of the Pacific non dipole low. J Geophys Res. 83(B4):1740–56. (1978).
15. P.A. Selkin, L. Tauxe, Long-term variations in palaeointensity. Phil. Trans. R. Soc., Lond. **358**, 1065–1088 (2000).

Captions to Figures and Tables

Supplementary Figure S1: Representative rock magnetic and palaeointensity results of flow # 9. (a) Temperature *versus* Susceptibility plot, (b) Isothermal remanent Magnetization curve, (C) Lowrie-Fuller test, (d) Arai plot along with orthogonal plot ; in the Arai plot (pTRM *vs* pNRM), red triangle refer to the pTRM checks, (e) normalized NRM decay curve; red square refer to the pTRM tail checks.

Supplementary Table S1: Palaeointensity results of the Deccan lava flows recovered within the 1250m Koyna drill hole (KBH-7) of the Scientific Deep Drilling Project; T: temperature range used to calculate the palaeointensity estimate; n: number of points used on the best fit line; PI, palaeointensity value; SD, Standard deviation of palaeointensity value; f: fraction of NRM used in the slope calculation; g: gap factor (Coe *et al* (14); q: quality factor; β, standard error of the best-fit straight line to the slope; MAD: Maximum Angular Deviation; d(ck) indicates pTRM checks; DRAT: Difference Ratio of pTRM checks (15) ; The field values are given with their standard deviation. The weighted average field is calculated using the quality factor as a weight; the average VDM is calculated using the weighted average palaeointensity values. Samples in bold are not considered for mean calculation (details in text)

Supplementary Table S2: Compilation of mean VDM values of the 65-66 Ma Deccan and other Cretaceous data by Thellier and microwave method with pTRM checks meeting the same criteria adopted in this study for the Deccan basalt. The results include data from igneous whole rocks with reliable radiometric ages; SBG data are not included as they lack directional data apart from alterations described in text. The single flow data for 110 Ma from Mangolia is used in the compilation as the data are sparse within mid CNS period. Data from Central Kerala, India, although from limited number of samples, are included in compilation because the results are from different time units and are in agreement with other data for the time period. PINT denotes PINT database; N and n are compiled from original publications.


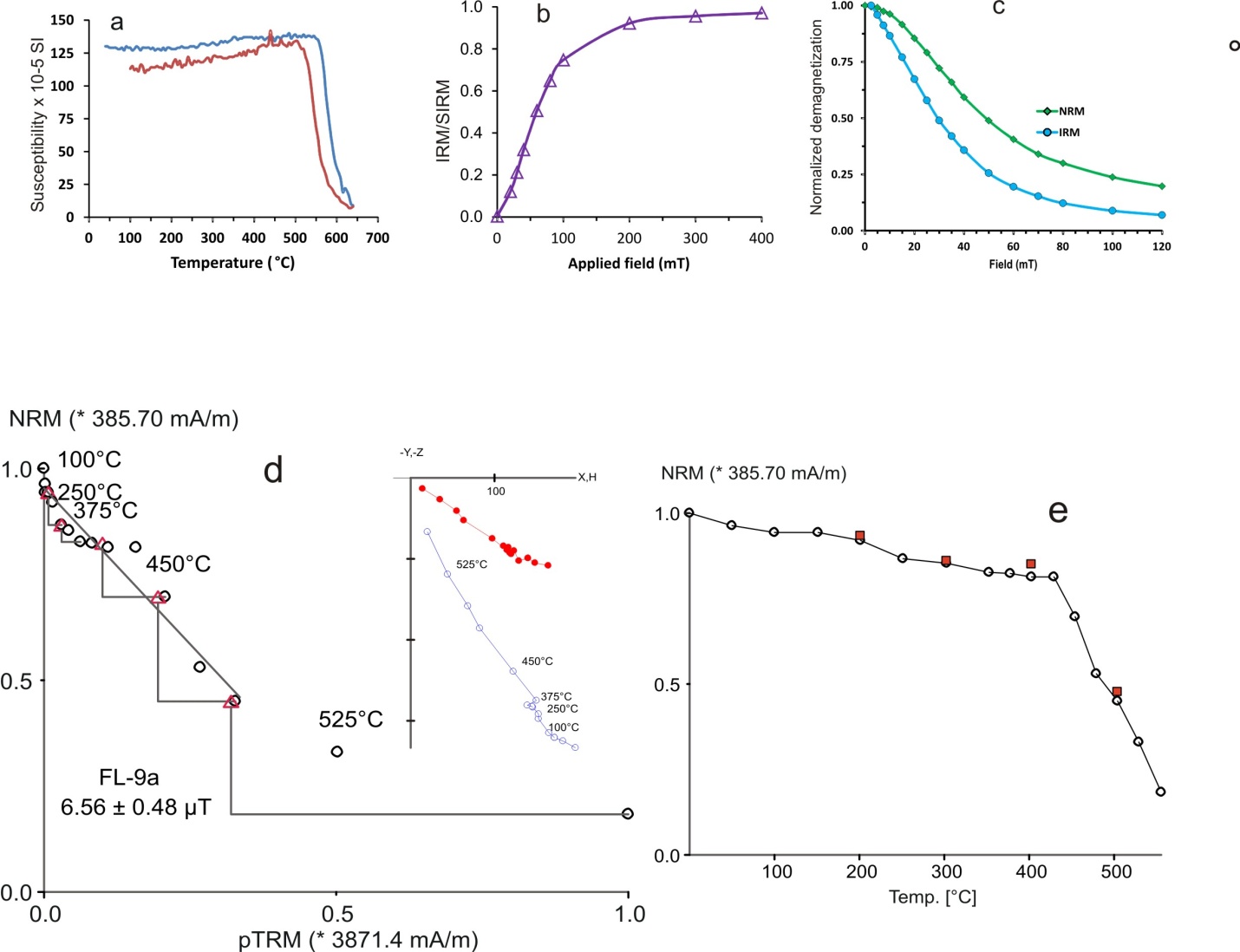


Supplementary Figure S1: Representative rock magnetic and palaeointensity results of flow # 9. (a) Temperature *versus* Susceptibility plot, (b) Isothermal remanent Magnetization curve, (C) Lowrie-Fuller test, (d) Arai plot along with orthogonal plot ; in the Arai plot (pTRM *vs* pNRM), red triangle refer to the pTRM checks, (e) normalized NRM decay curve; red square refer to the pTRM tail checks.

Supplementary Table S1: Palaeointensity results of the Deccan lava flows recovered within the 1250m Koyna drill hole (KBH-7) of the Scientific Deep Drilling Project.

| Lava flows | Samples | Tmin | Tmax | N | PI (µT) | SD (µT) | f | g | q | Β | MAD | d(ck) | DRAT | VDM X10E+22 |
| --- | --- | --- | --- | --- | --- | --- | --- | --- | --- | --- | --- | --- | --- | --- |
|  |  |  |  |  |  |  |  |  |  |  |  |  |  |  |
| Flow#1 | 1a | 150 | 500 | 11 | 10.43 | 0.82 | 0.5 | 0.87 | 5.6 | 0.08 | 4.2 | 3.6 | 6.9 | 2.1E+22 |
|  | 1b | 150 | 500 | 11 | 9.44 | 0.89 | 0.6 | 0.86 | 5.5 | 0.09 | 6.1 | 7.6 | 12.4 | 1.9E+22 |
|  | **1c** | **50** | **550** | **15** | **6.01** | **1.19** | **0.85** | **0.57** | **2.4** | **0.2** | **4** | **5.8** | **6.8** | **1.2E+22** |
|  | 1d | 50 | 475 | 12 | 11.46 | 1.34 | 0.40 | 0.87 | 3.0 | 0.12 | 3.3 | 2.5 | 6.1 | 2.3E+22 |
| Mean |  |  |  |  | 10.44±1.01 |  |  |  |  |  |  |  |  | 2.09±0.21 |
| Flow#2 | **2a** | **400** | **525** | **6** | **1.76** | **0.26** | **0.48** | **0.61** | **2** | **0.15** | **5.8** | **4.3** | **8.9** | **3.5E+21** |
| Flow#3 | **3a** | **375** | **550** | **8** | **1.29** | **0.17** | **0.93** | **0.62** | **4.3** | **0.13** | **6.9** | **7.2** | **7.8** | **2.6E+21** |
| Mean |  |  |  |  | *** |  |  |  |  |  |  |  |  | *** |
| Flow#8 | 8a | 100 | 475 | 11 | 11.53 | 1.03 | 0.69 | 0.86 | 6.7 | 0.09 | 1.4 | 4.1 | 5.8 | 2.3E+22 |
|  | 8b | 100 | 450 | 10 | 11.1 | 1.09 | 0.60 | 0.86 | 5.2 | 0.10 | 1.3 | 3.1 | 5.1 | 2.2E+22 |
|  | **8c** | **50** | **350** | **7** | **10.64** | **1.94** | **0.46** | **0.78** | **2.0** | **0.18** | **1.9** | **0.2** | **0.4** | **2.1E+22** |
|  | 8d | 50 | 450 | 11 | 10.2 | 0.78 | 0.57 | 0.87 | 6.5 | 0.08 | 1.7 | 3.3 | 5.5 | 2.0E+22 |
| Mean |  |  |  |  | 10.94±0.68 |  |  |  |  |  |  |  |  | 2.19±0.14 |
| Flow#9 | 9a | 50 | 500 | 13 | 6.56 | 0.48 | 0.52 | 0.85 | 6.0 | 0.07 | 1.3 | 2.9 | 5.6 | 1.3E+22 |
|  | 9b | 50 | 475 | 12 | 6.63 | 0.54 | 0.43 | 0.81 | 4.3 | 0.08 | 1 | 2.9 | 6.5 | 1.3E+22 |
|  | **9c** | **100** | **475** | **11** | **6.86** | **0.67** | **0.41** | **0.81** | **3.5** | **0.10** | **1.1** | **2.4** | **5.7** | **1.4E+22** |
|  | 9d | 50 | 475 | 12 | 6.56 | 0.56 | 0.43 | 0.82 | 4.2 | 0.08 | 1.5 | 2.9 | 6.7 | 1.3E+22 |
| Mean |  |  |  |  | 6.58±0.04 |  |  |  |  |  |  |  |  | 1.32±0.008 |
| Flow#14 | 14a | 50 | 550 | 15 | 2.96 | 0.11 | 0.65 | 0.67 | 11.3 | 0.04 | 2.2 | 2 | 3.1 | 5.9E+21 |
|  | 14b | 50 | 550 | 15 | 2.99 | 0.1 | 0.76 | 0.75 | 16.5 | 0.03 | 1.6 | 1.9 | 2.5 | 6.0E+21 |
|  | **14c** | **350** | **550** | **8** | **5.13** | **0.95** | **0.75** | **0.74** | **3.0** | **0.19** | **15.4** | **2** | **2.6** | **1.0E+22** |
|  | 14d | 50 | 550 | 15 | 3.37 | 0.21 | 0.63 | 0.80 | 7.9 | 0.06 | 1.7 | 2.1 | 3.3 | 6.8E+21 |
| Mean |  |  |  |  | 3.11±0.23 |  |  |  |  |  |  |  |  | 0.62±0.04 |
| Flow#16 | 16a | 100 | 425 | 9 | 8.33 | 0.47 | 0.42 | 0.81 | 6.0 | 0.06 | 2.7 | 2.6 | 6.2 | 1.7E+22 |
|  | 16b | 50 | 425 | 10 | 8.68 | 0.6 | 0.48 | 0.84 | 5.8 | 0.07 | 2.7 | 2.3 | 4.8 | 1.7E+22 |
|  | 18c | 50 | 425 | 10 | 8.68 | 0.64 | 0.49 | 0.83 | 5.5 | 0.07 | 4.3 | 2 | 4.1 | 1.7E+22 |
|  | **18d** | **100** | **450** | **10** | **6.76** | **0.75** | **0.45** | **0.84** | **3.4** | **0.11** | **4.4** | **1.9** | **4.3** | **1.4E+22** |
| Mean |  |  |  |  | 8.56±0.20 |  |  |  |  |  |  |  |  | 1.72±0.04 |
| Flow#19 | 19a | 50 | 525 | 14 | 1.49 | 0.12 | 0.82 | 0.66 | 6.6 | 0.08 | 2.3 | 2.4 | 2.9 | 3.0E+21 |
|  | **19b** | **50** | **475** | **11** | **7.16** | **0.89** | **0.32** | **0.41** | **1.1** | **0.12** | **2** | **0** | **0** | **1.4E+22** |
|  | 19c | 100 | 550 | 14 | 1.4 | 0.13 | 0.88 | 0.72 | 6.8 | 0.09 | 2.7 | 4.5 | 5.1 | 2.8E+21 |
|  | 19d | 100 | 550 | 14 | 1.29 | 0.12 | 0.89 | 0.73 | 6.8 | 0.1 | 4.8 | 6.9 | 7.7 | 2.6E+21 |
| Mean |  |  |  |  | 1.39±0.10 |  |  |  |  |  |  |  |  | 0.28±0.02 |
| Flow#22 | 22a | 100 | 475 | 11 | 8.16 | 0.41 | 0.32 | 0.87 | 5.6 | 0.05 | 2.4 | 3 | 9.1 | 1.6E+22 |
|  | 22b | 100 | 475 | 9 | 7.81 | 0.45 | 0.38 | 0.79 | 5.1 | 0.06 | 1.9 | 1.8 | 4.8 | 1.6E+22 |
|  | 22c | 100 | 475 | 10 | 8.52 | 0.9 | 0.57 | 0.74 | 4.0 | 0.11 | 2.5 | 2.6 | 4.5 | 1.7E+22 |
|  | 22d | 50 | 475 | 11 | 6.66 | 0.47 | 0.46 | 0.75 | 5.0 | 0.07 | 3.3 | 1.8 | 3.8 | 1.3E+22 |
| Mean |  |  |  |  | 7.79±081 |  |  |  |  |  |  |  |  | 1.56±0.16 |
| Flow#24 | 24a | 100 | 475 | 11 | 8.94 | 0.29 | 0.41 | 0.83 | 10.6 | 0.03 | 1.9 | 0.8 | 1.8 | 1.8E+22 |
|  | 24b | 100 | 475 | 11 | 8.29 | 0.24 | 0.42 | 0.82 | 11.7 | 0.03 | 1.9 | 0.6 | 1.5 | 1.7E+22 |
|  | **24c** | **375** | **550** | **8** | **26.83** | **4.88** | **0.99** | **0.7** | **3.8** | **0.18** | **1.1** | **2.3** | **2** | **5.4E+22** |
|  | 24d | 100 | 475 | 11 | 11.57 | 0.48 | 0.41 | 0.82 | 8.1 | 0.04 | 1.5 | 1.1 | 2.7 | 2.3E+22 |
| Grand Mean (N=8 flows) | |  |  |  | 7.30±3.45 |  |  |  |  |  |  |  |  | 1.46±0.69 |

T: temperature range used to calculate the palaeointensity estimate; N: number of points used on the best fit line; PI, palaeointensity value; SD, Standard deviation of palaeointensity value; f: fraction of NRM used in the slope calculation; g: gap factor Coe *et al* (14); q: quality factor; β, standard error of the best-fit straight line to the slope; MAD: Maximum Angular Deviation; d(ck) indicates pTRM checks; DRAT: Difference Ratio of pTRM checks (15); The field values are given with their standard deviation. The weighted average field is calculated using the quality factor as a weight; the average VDM (unit = 10^22^Am^2^) is calculated using the weighted average palaeointensity values.

Supplementary Table S2-: Compilation of mean VDM values of the 65-66 Ma Deccan and other Cretaceous data by Thellier and microwave method with pTRM checks meeting the same criteria adopted in this study for the Deccan basalt. The results include data from igneous whole rocks with reliable radiometric ages; SBG data are not included as they lack directional data apart from alterations described in text. The single flow data for 110 Ma from Mangolia is used in the compilation as the data are sparse within mid CNS period. Data from Central Kerala, India, although from limited number of samples, are included in compilation because the results are from different time units and are in agreement with other data for the time period.

|  | Fm/location | **Age** | **SD** | VDM E+22Am^2^ | **SD** | **N** | **n** | Ref |
| --- | --- | --- | --- | --- | --- | --- | --- | --- |
| 1 | Etendeka, Namibia | 133 | 1.0 | 2.5 | 1.00 | 5 | 64 | 10 |
| 2 | Parana, Brazil | 133 | 1.0 | 4.2 | 0.10 | 6 | 30 | PINT |
| 3 | Parana, Brazil | 132.5 | 0.5 | 7.2 | 2.30 | 10 | 42 | PINT |
| 4 | ArapeyFm, Parana, Uruguay | 132.2 | 0.5 | 6.4 | 2.30 | 5 | 27 | PINT |
| 5 | Parana, Argentina | 132 | 5.0 | 8.4 | 1.58 | 4 | 13 | PINT |
| 6 | Ponta Grossa Dikes, Brazil | 131 | 2.6 | 4.1 | 1.60 | 3 | 10 | PINT |
| 7 | Cordoba volc, Argentina | 130 | 5.0 | 6.70 | 1.61 | 5 | 21 | PINT |
| 8 | Liaoning province, China. | 120.93 | 0.9 | 3.96 | 0.07 | 11 | 42 | PINT |
| 9 | Liaoning province, China. | 119.3 | 1.2 | 3.5 | 0.06 | 3 | 16 | PINT |
| 10 | Jianguo, (Liaoning Province | 116.8 | 3.0 | 4.2 | 0.10 | 3 | 12 | PINT |
| 11 | Rajmahalvolc, India | 114.5 | 1.5 | 12.5 | 1.4 | 8 | 56 | PINT |
| 12 | Suhongtu Inner Mongolia | 114.1 | 0.3 | 4.61 | 2.61 | 3 | 20 | PINT |
| 13 | Suhongtu Inner Mongolia | 110.6 | 0.1 | 5.38 | 2.06 | 12 | 114 | PINT |
| 14 | Suhongtu Inner Mongolia | 110.6 | 0.1 | 5.18 | 1.87 | 11 | 44 | PINT |
| 15 | Mongolia | 110 | 5.0 | 7.7 | 1.40 | 1 | 7 | PINT |
| 16 | Strand Fiord Fm, Canada | 93 | 2.0 | 12.7 | 0.07 | 8 | 51 | PINT |
| 17 | Cyprus Ophiolite gabbro | 91 | 1.0 | 5.4 | 2.00 | 18 | 80 | PINT |
| 18 | Inner Mongolia | 91.5 | 5.5 | 3.2 | 1.60 | 6 | 46 | PINT |
| 19 | MtavariFom, Georgia | 85 | 15.0 | 3.3 | 0.10 | 4 | 29 | 11 |
| 20 | Gongju Basin, Korea | 77 | 3.4 | 3.51 | 1.26 | 3 | 31 | 12 |
| 21 | Yeoncheovolc, S. Korea | 76 | 5.0 | 2.98 | 0.70 | 4 | 14 | 13 |
| 22 | Central Kerala dol, India | 69 | 1.0 | 3.7 | 1.00 | 4 | 6 | PINT |
| 23 | Autlanvolc, Mexico | 67.4 | 1.2 | 4.9 | 0.60 | 4 | 18 | PINT |
| 24 | Deccan volc, India | 65.5 | 1.0 | 1.46 | 0.69 | 8 | 26 | This study |

SD denotes standard deviation; N and n denote number of flows/sites and number of samples used for mean values. Ref: reference number is linked to the references in the supplementary PINT denotes PINT database; N and n are compiled from original publications.
